# Flat-of-the-curve medicine: a new perspective on the production of health

**DOI:** 10.1186/2191-1991-1-2

**Published:** 2011-07-20

**Authors:** Johannes Schoder, Peter Zweifel

**Affiliations:** 1 Department of Economics, University of Zurich, Hottingerstrasse 10, CH-8032 Zurich, Switzerland

**Keywords:** production of health, control over health status, willingness to pay

## Abstract

Health economists have studied the determinants of the expected value of health status as a function of medical and non-medical inputs, often finding small marginal effects of the former. However, medical inputs may have an additional benefit in the form of a reduced variability of health status. Using the standard deviation of life expectancy in 24 OECD countries between 1960 and 2005, a 10 percent increase of health care expenditure is associated with a decrease of an estimated 0.42 percent. Willingness to pay for such a reduction of uncertainty may well exceed the extra health care expenditure in the United States and Switzerland. This implies that even in these two countries with very high health care expenditure per capita, flat-of-the-curve medicine need not be wasteful.

*JEL-Classification*: I12, J10

## Introduction

Industrial countries have been spending a rising share of their economic resources on health care. From 1960 to 2004 health care expenditure (HCE) of OECD countries increased from 3.8 percent of GDP to 8.9 percent on average. Over the same period, health outcomes measured by average life expectancy at birth improved from 68.4 to 78.5 years. However, this increase has slowed recently. In the United States e.g., it has been 0.19 percent p.a. between 1980 and 2004, down from 0.3 between 1960 and 1980. Since HCE continued to grow at a rate of 7.7 percent p.a. between 1980 and 2004, this has often been interpreted as evidence of decreasing marginal returns ("flat-of-the-curve medicine"; [1,2]), raising the question of why citizens and governments failed to reallocate resources away from medicine.

However, calling for such reallocation may be premature on at least two accounts. First, several studies based on individual health care expenditure data find that marginal returns to HCE still outweigh its marginal cost (see e.g. [3-5]; for an overview see [6]). This would explain why, in countries where individual willingness to pay for medical services tends to prevail over political considerations of cost control (such as the United States, the Netherlands, and Switzerland), the share of HCE in the GDP keeps increasing. Second, the implicit assumption that individuals only value changes in the expected value of health status is open to criticism. If people are risk-averse with regard to their health, they value a reduction in the variance of health status even if its expected value does not change. Thus, judging the benefits of HCE by its marginal product in terms of expected health (as traditionally done in studies of the production of health) possibly neglects the willingness to pay of risk-averse individuals for reduced uncertainty surrounding their health status.

Following up on this second aspect, this study seeks to determine whether the marginal cost in terms of HCE matches its marginal benefit if the reduction in uncertainty surrounding life expectancy is accounted for. In order to do this, we will proceed as follows. First, a conventional production function with life expectancy as the dependent variable is reestimated to verify that the countries of our sample are characterized by flat-of-the-curve medicine. Second, we examine whether a reduction of uncertainty surrounding life expectancy indeed occurred over the past 46 years. Third, based on the econometric estimation of an appropriately modified health production function we determine the relative contribution of medical and non-medical factors to reduced uncertainty. Finally, we compare the marginal cost of HCE with its marginal benefit in terms of willingness to pay for reduced uncertainty.

We find HCE as well as GDP to be significant determinants of the variance of health status. A 10 percent increase of HCE is estimated to lead to a 0.42 percent reduction of the standard deviation of life years. Furthermore, according to our calculations willingness to pay both in the United States and Switzerland for such a reduction exceeds the extra HCE, implying that additional HCE may be worth its cost as "real insurance", bringing back health status to normal when illness strikes. Hence, flat-of-the-curve medicine need not be wasteful.

Our study is closely related to the empirical literature on the production of health which uses aggregated health care expenditure data (e.g. [7-10], and [11]). Aggregated data have the advantage of providing panel measurements with variation between countries. However, rather than assessing the contribution of inputs exclusively to the expected value of health status, this study estimates their impact on the variability of health status as well. Furthermore, this work complements studies conducted on the individual level deriving willingness-to-pay values for health risk reductions either from experiments (see e.g. [12]) or from utility-theoretic models (see e.g. [13]).

## Theoretical background

**Figure 1:**
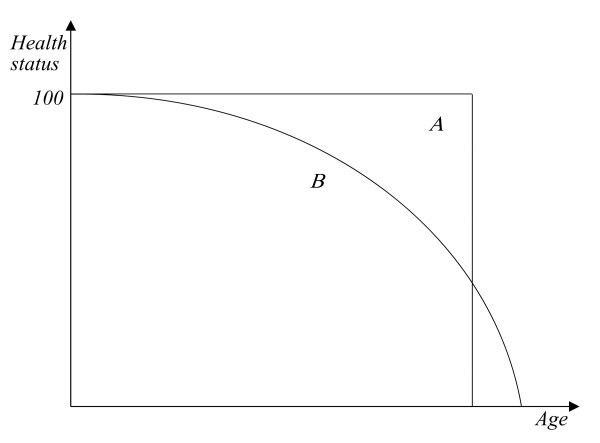
**Ranking of two health profiles**.

The basic hypothesis underlying this work is that individuals have preferences with regard to health profiles that are reflected in survival curves and their development over time. As a convenient starting point, consider the two hypothetical health profiles of Figure 1. Health profile A presumably represents the ideal of western lifestyle, living in perfect health followed by sudden death, indicated by a health status of zero ([14]). In contrast, health profile B represents an alternative where health status deteriorates with age but remains positive up to a higher age, indicating survival. The two profiles can also be interpreted as reflecting the probability of being in perfect health, which starts at 100 percent and stays there (profile A) or decreases with age (profile B). Thus, they represent cumulative distribution functions (cdfs), defined over the absence of death rather than (say) wealth. Even if profile B should have higher expected value, they can still be ranked in terms of second-order stochastic dominance (see e.g [15], ch. 2.5). In the present case, the triangle-like area above profile B (indicating the cumulative difference between the two cdfs) exceeds the extra area below profile B. In this event, an individual who is risk-averse with regard to health status prefers profile A. He or she has a positive willingness-to-pay (WTP) for living in a country with a health profile A rather than B.

Health profiles of this type are not available at this time. However, if individuals are successful in moving from profile B to the more rectangular profile A, they should in the aggregate exhibit an increasingly rectangular survival curve because survival constitutes the necessary (but not sufficient) condition for being in perfect health. Therefore, variability of age at death will be used as an indicator of uncertainty surrounding health status.

**Figure 2:**
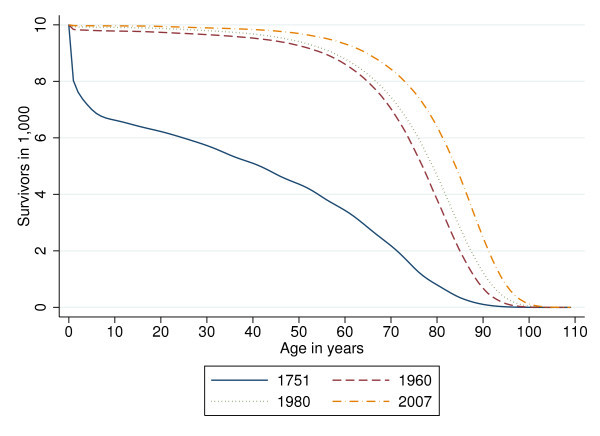
**Rectangularization of the survival curve in Sweden**.

Various indicators of variability of age at death (VAD) are used in the literature such as the interquartile range ([16]), the Gini coefficient ([17] and [18]), and the standard deviation ([19]). Regardless of choice of indicator, these studies document a secular decline in VAD for industrial countries, albeit at a somewhat reduced rate during the most recent decades. This development is tantamount to a rectangularization of the survival curve (see Figure 2 showing the case of Sweden). In keeping with the argument above, it is interpreted as evidence of individual's improved control over their health status. Note also that this improvement increasingly is reflected in the neighborhood of the nearly vertical segment of the nearly rectangular survival curve, calling for a special focus on the VAD of the elderly.

What this evidence is silent about is how individuals might have achieved this added control. In analogy with the production of health literature, it would be interesting to know whether the major contribution came from medical or non-medical inputs. To the authors' knowledge, there is only one study that relates a measure of VAD to medical and non-medical inputs ([20]). Due to data limitations, [20] performs but a cross-sectional regression for 1982 including 17 OECD countries. He relates public HCE, total HCE, GDP per capita, and a measure of income inequality (the share of the bottom quintile in national income) to the Gini coefficient of mortality. Public HCE has the expected negative impact on VAD but remains insignificant. Surprisingly, total HCE has a positive impact, whereas a higher GDP per capita and less income inequality are associated with lower VAD. However, the Gini coefficient is not translation independent (i.e. it changes if mean age at death differs between two periods or countries although the absolute differences between individuals' ages at death are the same). As to the interquartile range, it violates the transfer principle (it ignores a change in the distribution of deaths within a given age class if the number of deaths in that class does not change). For this reason, the standard deviation will be used as an indicator of VAD in the analysis below.

The present study extends previous work in three ways. First, it uses panel data tracing 24 OECD countries^a ^over the past 46 years, permitting to test the robustness of the results found by [20]. Second, since the relative contribution of medical and non-medical inputs may well change with age, VAD among the elderly (where rectangularization of the survival curve has been especially marked, see Figure 2) is examined in particular. Third, using evidence on the willingness to pay (WTP) for health risk reduction, WTP values are calculated for the two countries with the highest HCE per capita, the United States and Switzerland. These values are compared with the extra HCE to determine whether flat-of-the-curve medicine may be still worthwhile thanks to its effect on VAD. However, to address these research questions we first have to determine whether the countries of our sample indeed operate (on average) on the flat-of-the-curve. In sum, this leads to the following four research questions:

Q1: Are the countries of our sample characterized by flat-of-the-curve medicine?

Q2: Do medical or non-medical inputs contribute more to reducing variability of age at death (VAD)?

Q3: Are these effects different for VAD among the elderly?

Q4: Is flat-of-the-curve medicine wasteful?

## Methods

One way to assess the contribution of a set of inputs to remaining life expectancy at age 60 (*LE*_60_) and variability of age at death (VAD) is by eliminating certain causes of death from the data (see [17,16], but also [21], and [22]). However, comparing different countries over time entails the problem that this contribution may be conditioned by country-specific characteristics (e.g. the type of health care system). In contrast, econometric techniques designed for panel data permit to control for heterogeneity between countries either through fixed or random effects. In the fixed effects (FE) approach, the country-specific effects, *c*_*i*_, are included in the set of independent variables as a set of country-specific dummies. Alternatively, the *c*_*i *_can be netted out by measuring all variables as differences from their country-specific means. The random effects (RE) approach assumes the *c*_*i *_to be stochastic, which means they must be uncorrelated with the independent variables for unbiased parameter estimation. Both the RE and FE estimation were found to suffer from heteroskedasticity, reflecting cross-sectional correlation of error terms in Eqs. (4) and (5) below. Correcting for heteroskedasticity with a first-order autoregressive error term [AR(1) process] and applying the Hausman test we find that RE is preferred over FE throughout at the 5 percent significance level or better.

However, three additional issues need to be clarified. First, medical and non-medical inputs were found to influence remaining life expectancy with a lag by [11]. The same may be true for our sample. Alcohol consumption, for instance, likely does not undermine control over one's health immediately but rather over the course of years. Likewise, earlier HCE may also contribute to higher remaining life expectancy and lower VAD, respectively. As to GDP, it is interpreted as a budget constraint and an indicator of individual productivity. In principle, it could influence VAD with a lag as well. However, lagged GDP caused severe multicollinearity with lagged HCE, forcing a simplified specification with current GDP. Based on the Hausman test, we choose an optimal lag length of 10 years for HCE and ALC in Eq. (4) and 5 years for HCE in Eq. (5), values that seem to be reasonable in view of earlier research. Second, realizing a health profile as shown in Figure 1 calls for investment in (the control of) health over the life-cycle in response to age-dependent mortality. However, the corresponding health production function would require life-cycle measurements of HCE and other inputs, which are not available internationally at this time.^b ^The third issue is endogeneity. Remaining life expectancy and VAD may feed back to HCE. Countries where individuals live shorter or face higher uncertainty with regard to longevity may spend more on HCE than countries where individuals live longer or face less uncertainty. Such a feedback would likely occur through the political process, in analogy to the feedback relationship found by [11]. However, the Durbin-Wu-Hausman test ([23] and [24]) for endogeneity does not reject the null hypothesis of exogeneity of HCE at the one percent level.

Based on the econometric specification of [20] and the conventional health production approach (see e.g. [11]) the following specification is estimated (note that the variables in Eq. (2) are in logarithms):(1)(2)

The dependent variables are,

• *LE*_60_: Remaining life expectancy at age 60, corresponding to the average actual retirement age.

• *VAD*: Variability of age at death of country *i *in year *t *measured by the overall sd and the sd above the modal year, calculated according to Eqs. (4), (5).

Both variables are calculated from period life tables as follows (this type of life tables is discussed at the beginning of the next section). *LE*_60 _is the weighted average of age at death above 60, *x *- 60, with weights given by the number of individuals dying at the respective age, *d_x_*, relative to the number of survivors at age 60, *l*_60_(3)

where *ω *is the maximum age in the life table. This variable will be used to check whether industrial countries indeed are on the flat-of-the-curve with regard to HCE (see the section denoted "checking for flat-of-the-curve medicine").

The overall sd of age at death is given by(4)

with *le *symbolizing life expectancy at birth. In keeping with Figure 1, perfect rectangularization means a vertical drop of the health (and hence survival) profile. The age at which the greatest number of a cohort's members die approximates best this vertical drop. Therefore, the standard deviation above the modal year will be used to measure compression of mortality, which increasingly occurs at higher ages (see the example of Sweden again in Figure 1). The sd above the modal year is calculated in analogy to the overall sd,(5)

The independent variables are,

• *HCE*_-5/-10_: Total private and public HCE per capita, nominal but converted in 1,000 USD. Devoting more resources to health care is expected to enhance control over health status, and hence higher *LE*_60 _and lower VAD, repectively. Therefore, *α*_1 _is predicted to be positive and *β*_1 _to be negative.

• *GDP *: GDP per capita in 1,000 USD, nominal but converted in 1,000 USD. This variable first of all reflects the budget contraint. Now, length of life and control over one's health status is quite likely a normal good, the demand for which increases with average income, ceteris paribus. Second, however, average income is importantly determined by labor productivity. To the extent that non-market and market productivity develop in a similar way, a higher value of GDP reflects a population that is better able to control their health status. In this way, GDP also serves as an overall indicator of non-medical inputs. Both arguments suggest a positive sign for *α*_3 _and a negative sign for *β*_3_.

• *ALC*_-10_: Annual consumption of pure alcohol in liters per person above the age of 15. Lower values indicate a healthier lifestyle implying improved health and control over health status. Hence, *α*_5 _is predicted to be positive and *β*_5 _to be negative.

• *c*_*i*_: A set of country-specific dummies.

• *γ_t_*: A set of year-specific dummies to control for a possible time trend.

• *u_it_*: A stochastic error term, assumed to be i.i.d. normal.

### Data

Data to compute the dependent variables are obtained from the [25]. The Human Mortality database provides two different variants of life tables, cohort and period life tables. The former represent the mortality experience of individuals who are born in the same year and thus are truly comparable. However, cohort life tables contain complete mortality information only on cohorts without any survivors left. By way of contrast, period life tables show the estimated number of survivors at age *x *if a hypothetical birth cohort of 100,000 born today have the mortality rates that are observed today for people at various ages up to *x *([25]).^c^

**Figure 3:**
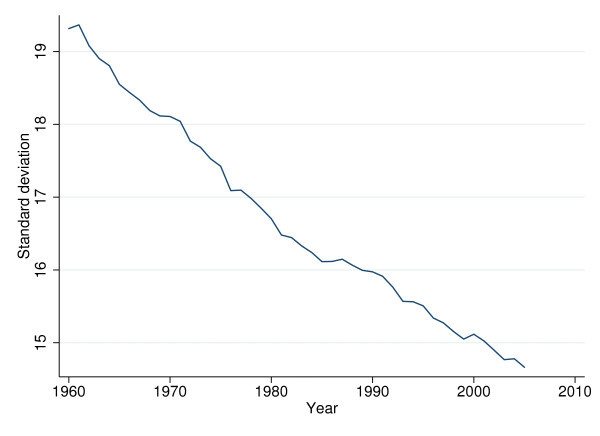
**Standard deviation of age at death averaged over 24 OECD countries, 1960 to 2005**.

**Figure 4:**
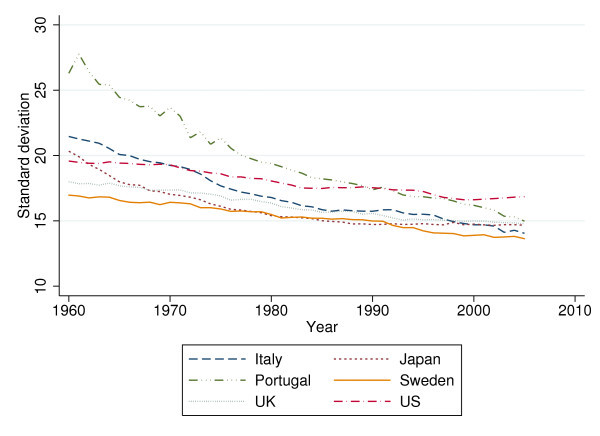
**Standard deviation for selected countries, 1960 to 2005**.

The overall sd exhibits an almost linear downward trend between 1960 and 1995 when averaged over 24 OECD countries (see Figure 3). Figure 4 traces the overall sd for six selected countries. Interestingly, the ranking between countries changed over time. In 1960, the Italians, Portuguese, and Japanese faced higher uncertainty of age at death than U.S. citizens (up to 7 years in terms of sd). However, by 2005 Americans faced considerably higher VAD than the citizens of these countries (almost 3 years in terms of sd).

**Figure 5:**
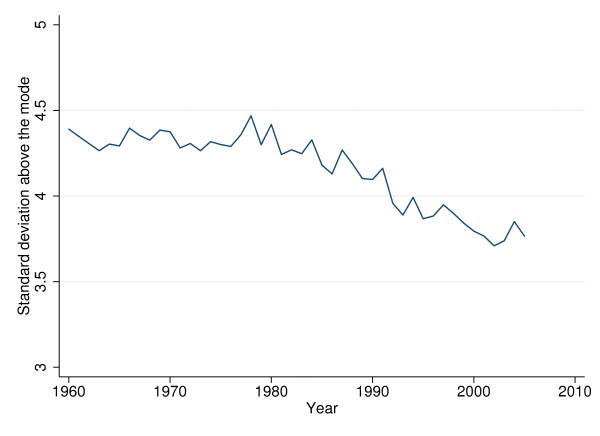
**Standard deviation above the modal year averaged over 24 OECD countries, 1960 to 2005**.

**Figure 6:**
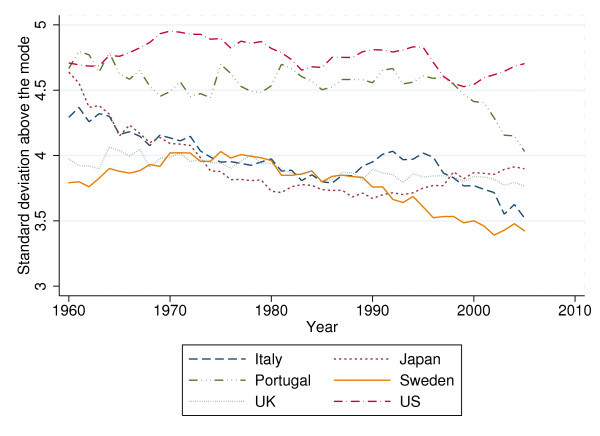
**Standard deviation above the modal year for selected countries, 1960 to 2005**.

The sd above the modal year exhibits a less regular pattern than the overall sd, mainly due to shifts in the age with maximum mortality. It remains roughly constant in the 1960s and 70s but has declined since 1980 (see Figure 5). Figure 6 shows that rectangularization at higher ages differs considerably between the six selected countries. It has declined for all six countries (except for the United States); however, the pattern of decline is again less regular than that of the overall sd.

In sum, the indicators for VAD confirm the findings of previous studies (see the section "theoretical back-ground"). They suggest that individuals in industrialized countries have been exposed to less uncertainty regarding their longevity (and presumably health status) since the 1960s, although differences between countries and subperiods (especially for the sd above the modal year) persist. The more the question of what may have contributed to these differential developments gains importance.

Turning to the independent variables, we defined medical and non-medical inputs drawing on the literature of the production of health ([7-10], and [11]). Due to missing values in the OECD health data base only the following determinants are retained. These are GDP per capita and alcohol consumption per capita in liters for the non-medical and health care expenditure per capita for the medical input. The OECD data is known for some problems. One of them is national differences with regard to the delimitation of the health care sector, resulting in different baskets of services, another the lack of comparability and precision of health care deflators. In the case of countries such as Switzerland or the Netherlands, HCE covered by basic health insurance are termed private HCE although basic insurance is mandatory and regulated by the government.^d ^In view of these difficulties, HCE is not split into private and public HCE (contrary to [20]). Furthermore, HCE is not deflated using national price indexes but by the exchange rate when converting the figures into USD, thus avoiding PPP indicators that may contain additional measurement errors (see [26]).

The sample comprising the 24 countries is characterized in Table 1 below. As to sd, the indicator of VAD, it decreases from 19.34 years in 1960 to 14.74 years in 2005. Decomposition of sd suggests that within-country differences (*sd_w_*) are rather more important than between-country ones (*sd_b_*), indicating that decreases over time are the primary source of variation. Turning to the independent variables, one observes slower growth of HCE and GDP in recent years. Whereas total HCE and GDP per capita increased by factors of 13 and 8, respectively between 1960 and 1983, these factors decreased to 4.5 and 3.5 between 1983 and 2005. Interestingly, alcohol consumption per capita (ALC) increased first but has been diminishing after reaching a peak in the mid 1970s. As to the decomposition of the standard deviation of the independent variables, variation over time again exceeds the between standard deviation (*sd_b_*), except for alcohol consumption.

**Table 1:** Descriptive statistics of variables, selected years

Variable	Mean	1960	1983	2005	**sd***_**o**_*	**sd***_**b**_*	**sd***_**w**_*	N
*LE*_60_	19.67	17.63	19.51	22.85	1.99	1.06	1.69	997
*SD*	16.81	19.34	16.44	14.74	1.76	0.97	1.49	1,102
*SD_mode_*	4.17	4.34	4.33	3.81	0.39	0.19	0.35	1,102
*HCE*	1,283	60.25	792.28	3,436	1,187	636.22	1068	838
*GDP*	13,866	1,341	10,287	35,782	11,903	7,787	10,780	965
*ALC*	10.62	7.87	11.81	9.41	3.66	3.27	1.78	1,003

*Note: *The countries included are Australia, Austria, Belgium, Canada, Czech Republic, Denmark, Finland, France, Germany, Hungary, Iceland, Italy, Japan, Luxembourg, Netherlands, New Zealand, Norway, Portugal, Slovak Republic, Spain, Sweden, Switzerland, United Kingdom, United States.

## Results and discussion

### Checking for flat-of-the-curve medicine

Table 2 presents RE estimation results for (arithmetic) *LE*_60_. This choice of dependent variable permits a comparison with [11], who estimated a similar specification for women and men separately using a different sample^e^. In general, the estimated coefficients roughly correspond with these previously estimated values (see column entitled *female *of Table 2). In keeping with these earlier estimates, HCE exhibits decreasing marginal returns.

**Table 2:** Determinants of remaining life expectancy at age 60 (total population), 1960-2005

*LE*_60_	**Coef**.	z	P>z	Coef. (*female*)*^a^*
*HCE*_-10_	0.842	2.49	0.013	2.045**
	-0.199	-1.99	0.046	-0.565**
*GDP*	0.041	2.06	0.040	0.122**
*GDP*^2^	-0.001	-2.11	0.036	-0.004**
*ALC*_-10_	-0.039	-0.60	0.546	-0.043
	-0.002	-1.09	0.276	0.002
constant	17.230	51.14	0.000	18.57

*rho_ar_*	0.911			
Wald *χ*^2^	719.24			
Prob>*χ*^2^	0.814			
R-squared	0.477			
Observations	631			

Note: **p < 0.01.*^a^*Estimates for females from [11].

With regard to remaining life expectancy, the critical value of HCE beyond which its marginal effect ceases to be positive can be put at USD 2,116.^f ^With a mean value of USD 3,436 as of 2005 OECD countries on average are well within the flat-of-the-curve range. Therefore, as to research question Q1 stated at the beginning of this paper, we can conclude that the countries in our sample operate on the flat-of-the-curve.

However, this traditional view on the production of health may well neglect the impact of HCE on the uncertainty surrounding life expectancy. This is addressed in the following section.

### Variability of age at death as the dependent variable

Table 3 presents (double-log) RE estimation results for *sd *and *sd_mode_*, the two indicators of variability of age at death emphasized here. For a comparison with Table 2, elasticities of *LE*_60 _evaluated at the means are provided in the column entitled Table 2. Three things are noteworthy. First, the same inputs that were found to increase (decrease) the expected value are estimated to decrease (increase) the variability of life expectancy. Second, whereas GDP is more effective than HCE in increasing the expected value (see fifth column), it tends to be less effective in reducing its variability of longevity. Third, HCE exhibits decreasing returns also as an instrument for controlling variability of health status.

**Table 3:** Determinants of variability of age at death, 1960-2005

	*sd*			Table 2	*sd_mode_*		
			
VAD	**Coef**.	z	P>z	*ε* ***LE***_**60**_	**Coef**.	z	P>z
*HCE*_-5_	-0.072	-3.09	0.002	0.032	-0.019	-0.27	0.788
	0.005	2.65	0.008		0.002	0.28	0.783
*GDP*	-0.066	-1.99	0.046	0.041	-0.006	-0.03	0.980
*GDP*^2^	0.004	0.94	0.345		0.001	0.06	0.955
*ALC*_-10_	0.049	1.29	0.198	-0.029	0.210	2.17	0.030
	0.017	1.87	0.061		-0.046	-1.97	0.049
constant	3.435	11.47	0.000		1.321	1.43	0.154

*rho_ar_*	0.786				0.237		
Wald *χ*^2^	1,103				280.46		
Prob>*χ*^2^	0.000				0.000		
R-squared	0.6390				0.3407		
Observations	631				631		

Turning to the detailed estimation results for the overall standard deviation in Table 3, we find *HCE*_-5 _to be significant and with the predicted negative sign. Evaluated at the mean values, a 10 percent increase of HCE 5 years earlier is estimated to reduce the current standard deviation of age at death by  percent. The effect of non-medical inputs is in the same range, with an increase of GDP by 10 percent associated with a decrease of variability by 0.66 percent (neglecting the insignificant squared term). As predicted, an unhealthy lifestyle proxied by *ALC*_-10 _seems to weaken control over health status. An earlier increase of alcohol consumption by 10 percent increases VAD by an estimated 0.49 percent (again neglecting the squared term).

For the standard deviation above the modal year, the results are quite different. Only alcohol consumption is significant at the 5 percent level, with a similar estimated effect. A 10 percent increase 10 years earlier goes along with a  percent increase of the standard deviation above the modal year. Especially at older ages, unhealthy lifestyle in the past seems to induce a lack of control over health status. Still, the insignificant coefficients pertaining to HCE come as a surprise because according to e.g. [8], health status of the elderly (measured by their remaining life expectancy) appears to have strongly benefited from pharmaceutical innovation in particular. The apparent contradiction may be resolved by referring back to Figure 5. There, it appears that HCE may have influenced variability of age at death among the elderly only in recent years, possibly due to medical progress for the treatment of old-age diseases (e.g. circulatory and respiratory diseases and cancers; for some evidence, see [27]). The graph suggests reestimation of the model for the time period between 1983 to 2005. Results are presented in Table 4 below.

**Table 4:** Determinants of variability of age at death above the modal year (*sd_mode_*), 1983-2005

Explanatory variable	**Coef**.	z	P>z
*HCE*_-5_	-0.056	-2.49	0.013
	0.005	0.47	0.639
*GDP*	-0.058	-2.97	0.001
*GDP*^2^	0.002	0.15	0.881
*ALC*_-10_	0.061	0.28	0.776
	-0.017	-0.36	0.719
constant	1.774	5.52	0.000

*rho_ar_*	0.217		
Wald *χ*^2^	2,284		
Prob>*χ*^2^	0.000		
R-squared	0.2921		
Observations	430		

Now, *HCE*_-5 _turns out to be significant at the 5 percent level, with a 10 percent increase serving to reduce variability of age at death by an estimated 0.56 percent. Almost the same magnitude is found for GDP. However, *ALC*_-10 _is found to be insignificant. Also note that the estimated coefficients pertaining to HCE and GDP cannot be distinguished from those for the overall sd in Table 2. Therefore, as to the research question Q2, we can conclude that both medical and non-medical inputs contribute to the observed reduction in VAD, and to a comparable extent. As to research question Q3, the answer depends on the period of observation. For the period as a whole (1960 to 2005), the elderly seem to differ in that VAD above the modal year cannot be related to either HCE or GDP. However, from the mid-1980s on, these two variables have effects that are comparable to those on the general population.

### Is flat-of-the-curve medicine wasteful?

In the previous section, evidence was presented to the effect that many OECD countries presently are characterized by flat-of-the-curve medicine if the marginal contribution of HCE to remaining life expectancy is accepted as the criterion. However, according to the estimation results presented in Tables 3 and 4, HCE does contribute to reduced uncertainty with regard to health status. In the present context, this effect is valued using the risk premium an individual would be willing to pay for reducing the risk of premature death indicated by the variability of age at death (VAD).

Theoretically, the risk premium can be derived from the following equality condition that makes an individual indifferent between the certain health status A after deduction of the premium  and the risky health profile B of Figure 1. In Eq. (6) below, *u*[*H_A_*] denotes the certain utility associated with certain health A (in money equivalent) and *EU*, expected utility associated with risky health B, which is composed of *H_A _*and a small variation  of health status,(6)

Applying Taylor approximations to both sides and solving for , one obtains the Arrow-Pratt formula in terms of health rather than wealth (see [28], ch. 3)(7)

with  defining the coefficient of absolute risk aversion. The risk premium therefore is given by the product of (one half of) the variance of health status, , and the coefficient of absolute risk aversion. Now, risk aversion with regard to a variation in health may well differ from risk aversion with regard to wealth. Therefore, it is important to use an estimate that has a close connection to health. The one by [29] qualifies because it is derived from the choice of health insurance. His value of *R_A _*is 3·10^-3^; in the interest of a conservative estimate of the risk premium, we use a value of *R_A _*equal to 10^-4^. The next step is to express the variance of length of life (as an indicator of health), , in monetary units. This will be done for the two countries that devote very high per-capita amounts to health care and therefore likely constitute two extreme cases of flat-of-the-curve medicine, the United States and Switzerland. The United States Environmental Protection Agency ([30]) has been using a value of USD 6.3 mn. per statistical life in its cost-benefit analyses since 1999. [31] estimate an average value of CHF 12.5 mn. for Switzerland based on labor market data as of 1995. Taking the base year 2000 and an interest rate of 3 percent, this amounts to a value of a statistical life of USD 6.5 mn. for the United States and USD 8.7 mn. for Switzerland (with and an exchange rate of 1CHF = 0.6USD).

Given that the two estimates above relate to statistical lives and hence are the result of a linear extrapolation of small changes in survival probabilities, it is also admissible to interpret them as linear extrapolations of a change of one year of life expectancy. With average life expectancies of 73.8 years (United States) and 76.2 (Switzerland) respectively, one statistical year of life is worth USD 87,927 in the United States and USD 114,102 in Switzerland. Based on a utility-theoretic model of preferences over length of life, [13] predicts that an individual would be willing to trade one-half a year of additional life expectancy against a reduction of uncertainty by one standard deviation. Therefore, a change of one sd in age at death can be valued at some USD 43,963 (United States) and USD 57,051 (Switzerland), respectively. According to the estimation results presented in Table 3 a 10 percent increase of HCE is estimated to reduce the sd by 0.42 percent, i.e. from 18.01 to 17.93 years in the US and from 16.24 to 16.17 in Switzerland.

Inserting these estimates into Eq. (7) we obtain the following willingness-to-pay (WTP) values for such a reduction:(8)(9)

Distributed over 73.8 years this becomes a WTP value for the United States of USD 3,771 and USD 4,261 for Switzerland. The last step concerns the marginal cost. In 2000, the United States spent USD 4,704 per capita on health care and Switzerland, USD 3,529. Hence, 10 percent more HCE amounts to USD 470 and USD 353, respectively. The comparison with the estimates in Eqs. (8) and (9) clearly shows that in both countries, WTP for increased certainty with regard to age at death exceeds their marginal cost in terms of HCE. Therefore, one can answer Q4 by concluding that even if HCE should not prolong life anymore, it may be worth its cost as "real insurance" reducing the variability of health status.

## Conclusions

This study addresses an issue that has been overlooked in the production of health literature with its emphasis on flat-of-the-curve medicine. For risk-averse individuals, not only the level of health but also its variability is important. However, improved control over health status is reflected in an increased rectangularization of the survival curve, indicating a reduced variability of age at death (VAD). Since this rectangularization can indeed be observed in OECD countries, this raises four research questions. Are the countries of our sample characterized by flat-of-the-curve medicine (Q1)? Do medical or non-medical factors contribute more to reducing VAD (Q2)? Do these effects differ among the elderly, where rectangularization has been prominent (Q3)? Is flat-of-the-curve medicine wasteful (Q4)?

The standard deviation (sd) of age at death serves as an indicator of overall uncertainty concerning health status and the sd above the modal year (where the number of deaths in adulthood reaches its maximum) as an indicator of uncertainty surrounding health status among the elderly. Between 1960 and 2005 both measures decreased for the 24 OECD countries sampled, pointing to reduced VAD. However, sd above the modal year began to fall in the early 1980s only. These two indicators are related to HCE as a proxy of medical inputs to the production of health and to GDP and alcohol consumption as a proxy of non-medical ones. Based on a specification that takes account of hidden heterogeneity through random effects, the four research questions can be answered as follows.

Q1: According to our estimates the critical value of HCE beyond which its marginal effect ceases to be positive can be put at USD 2,116. With a mean value of USD 3,436 as of 2005, the OECD countries of our sample are on average well within the flat-of-the-curve range.

Q2: The reduction of VAD (indicating better control over health status) is importantly due to both, HCE and GDP.

Q3: Significant effects of HCE and GDP on VAD among the elderly are found for the time period between 1983 to 2005 only, of a magnitude comparable to Q2.

Q4: Comparing the marginal cost in terms of HCE with the willingness-to-pay values for the United States and Switzerland, we find that the benefits in terms of reduced VAD exceed the extra cost. Therefore, flat-of-the-curve medicine may be worthwhile as "real insurance" serving to reduce uncertainty of health status.

However, several limitations of this study need to be pointed out. First, variability of health status as experienced by individuals is only crudely measured by cross-sectional measures such as the standard deviation of age at death. Tracking individual's health status over time would be preferable, but availability of panel data would restrict the analysis to a few countries only. Second, medical and especially non-medical inputs to the production of health are not very well captured by HCE and GDP and alcohol consumption per capita, respectively. Unfortunately, measures of education and other indicators of lifestyle do not date back sufficiently far for many OECD countries. Third, we used a coefficient of absolute risk aversion derived from U.S. data. Its value likely differs between countries.

However, the findings on the whole do suggest that variability of health status can be influenced. This has important implications. First, reduced uncertainty about age at death likely has been modifying the decisions especially of older individuals concerning savings, consumptions, and the purchase of life and long-term care insurance. Quite generally, it helps risk-averse individuals to optimize lifetime consumption, permitting them to reduce precautionary saving (see [32] and [33]). Second, knowing the extent and determinants of variability of health status enables insurers and reinsurers to calculate more accurate values of the financial risk they are exposed to and expected to face in the future in different countries. Finally, our study suggests complementing the economic evaluation of medical interventions (such as cost-utility or cost-effectiveness analysis) with possible reductions in the uncertainty of outcomes.

## Abbreviations

FE: Fixed effects; HCE: Health care expenditure; RE: Random effects; VAD: Variability of age at death; WTP: Willingness to pay.

## Competing interests

The authors declare that they have no competing interests.

## Authors' contributions

Both authors have made substantial contributions to the intellectual content of this paper.

## Endnotes

^a^These are Australia, Austria, Belgium, Canada, Czech Republic, Denmark, Finland, France, Germany, Hungary, Iceland, Italy, Japan, Luxembourg, the Netherlands, New Zealand, Norway, Portugal, Slovak Republic, Spain, Sweden, Switzerland, United Kingdom, and the United States.

^b^We owe this important point to an anonymous referee.

^c^A disadvantage of period life tables is that they are based on one-year age intervals and that it is assumed that infants born today will experience the same mortality risks over their lifetimes as do the various age groups in the current population (see [18]). In times of decreasing mortality rates, period life tables underestimate life expectancy.

^d^E.g. premiums for the basic mandatory coverage are not risk-rated.

^e^They included consumption of kilocalories per capita as an additional lifestyle variable, which however turned out to be not significant.

^f^From Table 2, one obtains the critical value beyond which *e*(*LE, HCE*) decreases: . This yields *HCE *= 2.116 or 2,116 USD respectively, which is in the same range as the critical value estimated in [11].
